# Commentary: Interventricular Differences in Action Potential Duration Restitution Contribute to Dissimilar Ventricular Rhythms in *ex vivo* Perfused Hearts

**DOI:** 10.3389/fcvm.2019.00058

**Published:** 2019-05-13

**Authors:** Andreas Kucher, Roland X. Stroobandt

**Affiliations:** ^1^BIOTRONIK SE & Co. KG, Berlin, Germany; ^2^Department of Cardiology, Ghent University Hospital, Ghent, Belgium

**Keywords:** implantable cardioverter-defibrillator, cardiac resynchronization therapy (CRT), ventricular tachycardia, ventricular fibrillation, dissimilar ventricular rhythms, tachycardia detection, CRT-D, cardiac resynchronization therapy–defibrillator

Since the discovery of dissimilar ventricular tachyarrhythmias using implantable cardioverter defibrillators (ICD) with cardiac resynchronization therapy (CRT-D) with the capability of left ventricular electrogram (EGM) recording (1), it was postulated that dissimilar ventricular rhythms might exist predominantly with rate disparities where the left ventricular (LV) rate is faster than the right ventricular (RV) rate.

In a recently published article in this journal entitled “Interventricular Differences in Action Potential Duration Restitution Contribute to Dissimilar Ventricular Rhythms in *ex vivo* Perfused Hearts”, Handa et al. used rat hearts to investigate the inducibility of dissimilar rhythms, the impact of antiarrhythmic drugs and the underlying mechanism (2). The authors correctly defined dissimilar ventricular rhythms as the occurrence of different ventricular tachyarrhythmias in the right and left ventricles, or different rates of the same arrhythmia in the two ventricles.

In the different quoted publications, dissimilar ventricular rhythms typically occur with an LV rate that is faster than the RV rate (LV>RV), which can lead to serious complications in correct ICD detection and the delay or absence of therapy. Momentarily, the detection of ventricular tachyarrhythmias (VAs) is exclusively based on the implanted electrode in the RV. A correct ICD detection cannot be expected if the RV rate is below the programmed cut-off rate, although ventricular fibrillation (VF) exists in the left ventricle.

The results in the above mentioned study (2) have shown exclusively LV>RV scenarios. A possible suggestion that was mentioned, would be to change the detection from the right to the left side.

However, in other CRT-D recordings (Figure 1) we observed episodes whereby the RV rate was faster than the LV rate (RV>LV). A fixed and predefined detection side for the ICD would therefore not be useful as no one can predict the presence of dissimilar ventricular rhythms. It is also not possible to predict in which chamber the possibly faster VA rate exists. A new dynamic detection principle could be helpful, whereby the ICD can decide itself from which side the faster heart rate originated. The technical solution requires certain special safety concepts to prevent inaccurate detection of atrial fibrillation in the event the LV electrode is dislocated into the atrium. A dislocation of one of the ventricular leads into the atrium can be safely excluded with the help of the EGM tracing in Figure 1, even in the absence of an X-ray image for two reasons: 1. The atrial channel shows sinus rhythm. Therefore, atrial fibrillation being wrongly detected by a possibly dislocated ventricular electrode can be excluded. It is genuine VF in the RV-channel; 2. The A-RV-LV signal sequence directly after the shock suggests a perfectly positioned LV electrode. The RV and LV signals are sufficiently separated from each other, which show the interventricular conduction delay caused by the left bundle branch block. A dislocated LV electrode in the atrium would present a prematurely detected LV signal at a time close to the true atrial excitation.

**Figure 1:**
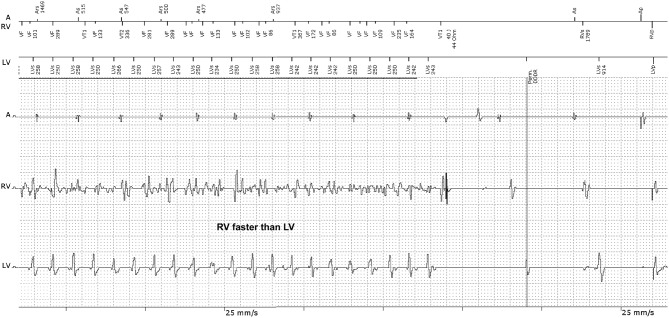
EGM recording of a CRT-D device with appropriate VF detection during dissimilar ventricular tachyarrhythmia. The RV-EGM shows typical morphologies of VF, whilst the LV-EGM shows RR intervals of a quasi-stable ventricular tachycardia (VT). The ICD recognized VF correctly because the ICD detection is based on the RV electrode and delivered a 40 Joule shock, restoring sinus rhythm. A, atrium; RV, right ventricle; LV, left ventricle.

Frequently, dissimilar ventricular rhythms will be triggered by anti-tachy pacing, intraoperative induction shocks or ineffective therapeutic shocks. It is rather rare that such heart rate disparities occur spontaneously. In the prehistory of the EGM recording (not shown in Figure 1), there was a previously ineffective shock that had initiated the dissimilar ventricular rhythm.

In our experience, the probability of discovering RV>LV is even higher than the incidence of LV>RV discovery, possibly because RV>LV can be better detected and treated by the ICD compared to LV>RV. A dynamic dual-side detection principle would certainly be an interesting idea. However, it can only be a possible concept in CRT-D devices. It is unfortunately not applicable in the single- or dual-chamber ICDs because of the absence of LV sensing (3, 4). Further technical investigations in favor of a functioning hemodynamic sensor are possibly more promising as this would be applicable in all types of ICDs. For the current generations of ICDs, the occurrence of RV>LV is less dramatic, as it will be recognized by the ICD.

It is remarkable that the phenomenon of RV>LV was not observed in the present study. Perhaps these cases of RV>LV could not be triggered due to the fact that this is an animal study and conditions other than those found in humans may prevail. The authors should be commended for their elegant study looking into the physiological mechanism of dissimilar rhythms, thereby lending support for clinical observations. It would be of great scientific interest if the authors can be encouraged to continue their efforts and expand their research to both observations of LV>RV as well as RV>LV, to reproduce them in the lab.

## Author Contributions

All authors listed have made a substantial, direct and intellectual contribution to the work, and approved it for publication.

### Conflict of Interest Statement

AK is an employee of BIOTRONIK SE & Co. KG. The remaining author declares that the research was conducted in the absence of any commercial or financial relationships that could be construed as a potential conflict of interest.
